# Multifunctional photon conversion materials for enhancing silicon solar cells

**DOI:** 10.1038/s41377-024-01431-3

**Published:** 2024-04-09

**Authors:** Yiyan Zhang, Guanying Chen

**Affiliations:** MIIT Key Laboratory of Critical Materials Technology for New Energy Conversion and Storage, School of Chemistry and Chemical Engineering, Harbin Institute of Technology, 150001 Harbin, China

**Keywords:** Nanoparticles, Fluorescence spectroscopy

## Abstract

A type of multifunctional erbium (Er^3+^) and ytterbium (Yb^3+^) codoped NaY(WO_4_)_2_ phosphors, with simultaneous photon upconversion, photon quantum cutting, and luminescence ratiometric temperature sensing abilities, have been developed, opening up new possibilities for high-performance silicon solar cells.

As traditional fuel sources (coal, oil, and natural gas) continue to deplete on a global scale, there is a surging demand for renewable energies. The Sun irradiates Earth with 120,000 terawatts of power, while humans consume only 15 terawatts globally. Solar cells have demonstrated significant potential by converting sunlight into electricity through the photovoltaic effect.

Silicon solar cells (SSCs), based on crystalline or polycrystalline silicon, dominate the world photovoltaic market, constituting ~95% of the total global production in 2022^1^. Despite their market dominance, the power conversion efficiency of industrially produced solar modules is within a marginally acceptable range of 18–22% under standard test conditions (Air Mass 1.5). The theoretical maximum efficiency for single-junction SSCs, with a bandgap (E_g_) of 1.1 eV, is ~30% according to the Shockley-Queisser limitation^2^. The primary challenge lies in the significant loss of 70% of Sun energy irradiated to the photovoltaic device, which is attributed to the spectral mismatch between the solar terrestrial spectrum and the absorption spectrum of silicon semiconductor. This mismatch involves thermalization relaxation of photoexcited ‘hot’ charge carriers (electron/hole) to the band edges (both the conduction band (for the electron) and the valence band (for the hole)) (thermalization loss) and the transmission of photons with energies below the bandgap of the silicon (transmission loss). In other words, spectral mismatch results in the under-utilization of harvested ultraviolet light (<530 nm, ~149 W m^−2^) as well as the non-utilization of transmitted infrared light (>1100 nm, ~164 W m^−2^)^3^. Advances in SSCs are urgently needed to increase the performance and reduce the cost of harvesting solar power.

Photon upconversion and quantum-cutting materials present a promising solution to address the spectral mismatch challenge in SSCs by effectively managing the solar spectrum. Upconversion involves the conversion of two or more sub-bandgap infrared photons into a single above-bandgap photon, generating useful photoexcited electron-hole pairs. A detailed balance model, as proposed by Trupke et al., predicts the efficiency limits of a solar cell with an upconverter placed beneath a bifacial solar cell and a perfect reflector at the upconverter’s rear surface. The calculated maximum efficiency reaches 50.69% at a bandgap (*E*_g_) of 2 eV and 40.2% at *E*_g_ = 1.1 eV (silicon bandgap) under unconcentrated terrestrial Air Mass 1.5 incident light, surpassing the Shockley-Queisser limitation^4^.

On the flip side, quantum cutting is a reverse process to upconversion, in which a high-energy photon is split into two (or more) lower-energy photons. The photoluminescence quantum yields of quantum cutting materials theoretically can exceed 200%, showcasing promise for applications in light-emitting diodes and solar cells^5–8^. For solar cells, this implies that one ultraviolet photon can produce two electron-hole pairs, thereby enhancing power conversion efficiency. Predictions indicate that mounting a photon quantum cutting layer on the front surface of a conventional single-junction SSCs could achieve a maximum power conversion efficiency of up to 38.6%, exceeding the Shockley-Queisser limitation^9,10^. Importantly, the application of spectral converting layers does not necessitate alterations to the existing solar cell device structure. As a result, there is a growing demand for phosphors with both upconversion and quantum cutting capabilities to address the spectral mismatch problem in current SSCs.

Furthermore, the in-situ temperature measurement of operating SSCs is imperative. This is particularly true due to the potential temperature escalation caused by high-intensity sunlight exposure, which may result in decreased photovoltaic output or even module failure^11^. Luminescence nanothermometry, especially the one based on the sharp luminescence of lanthanides, provides a means for precise monitoring of local heat dissipation. This monitoring occurs at high spatial resolution, reaching down to the nanoscale, and allows for remote detection without the need for physical contact between probes and detectors^12^.

The development of phosphors with upconversion and quantum cutting abilities, along with effective temperature probing, is therefore on demand to significantly enhance the performance of SSCs. This need was not successfully addressed until recently in a study published in Light: Science & Applications by Gao et al. where they introduced a multifunctional phosphor, erbium (Er^3+^), and ytterbium (Yb^3+^) doped NaY(WO_4_)_2_^13^.

Intense and nearly-pure emission from Yb^3+^ under 378 nm excitation was observed due to a concentration-dependent quantum cutting process, as depicted in Fig. 1. A set of singly Er^3+^-doped NaY(WO_4_)_2_ phoshpors experiments illustrated that photoexcitation at 378 nm, matching the ground sate absorption of ^2^I_15/2_ → ^4^G_11/2,_ exclusively produced intense 530/552 nm emissions at the ^2^H_11/2_/^4^S_3/2_ energy levels, respectively, with negligible emissions from the lower-lying ^4^F_9/2_ energy level. This observation indicates that the energy of absorbed photons within the energy levels between ^2^H_11/2/_^4^S_3/2_ and ^4^G_11/2_ can all be entirely accumulated to the ^2^H_11/2_/^4^S_3/2_ reservoir through multiphonon-assisted nonradiative relaxations. The cumulative addition of harvested ultraviolet photons to the appropriate energy level is a crucial step in maximizing the effectiveness of the photon quantum-cutting process. When fixing Er^3+^ dopant concentration at the optimized one (5%), codoping Yb^3+^ ions (from 0% to 50%) results a continuous increase of Yb^3+^ infrared luminescence at 1000 nm (^2^F_7/2_ → ^2^F_5/2_), coinciding with a progressive decrease of 530/552 nm luminescence from the ^2^H_11/2_/^4^S_3/2_ reservoir energy levels. In contrast, the emission from the ^2^H_9/2_ → ^4^I_9/2_ transition of Er^3+^, while weak, remains constant. These results validate the presence of efficient energy transfer (ET1) process, which depopulates the ^2^H_11/2_/^4^S_3/2_ energy levels of Er^3+^ and results in the simultaneous population of the ^2^F_5/2_ energy level of Yb^3+^ ions and the ^4^I_11/2_ energy level of Er^3+^ ions (Fig. 1). Radiative and nonradiative relaxations from the ^4^I_11/2_ energy level are expected to populate the ^4^I_13/2_ energy level, leading to amplified infrared emission at 1550 nm due to the ET1 process. However, a decrease in luminescence intensity at 1550 nm was observed, indicating the presence of the ET2 process. In this process, the Er^3+^ at the ^4^I_11/2_ energy level will transfer its excited energy to the ground state ^2^F_7/2_ of Yb^3+^, promoting the Yb^3+^ dopant to its excited ^2^F_7/2_ energy level. Judd-Ofelt calculations and experimental luminescence decay measurements were employed to elucidate the radiative rates, nonradiative rates, and energy transfer rates of all significant energy levels in Er^3+^ ions. These analyses unveiled an ultrahigh quantum cutting efficiency of 173%.

**Fig. 1:**
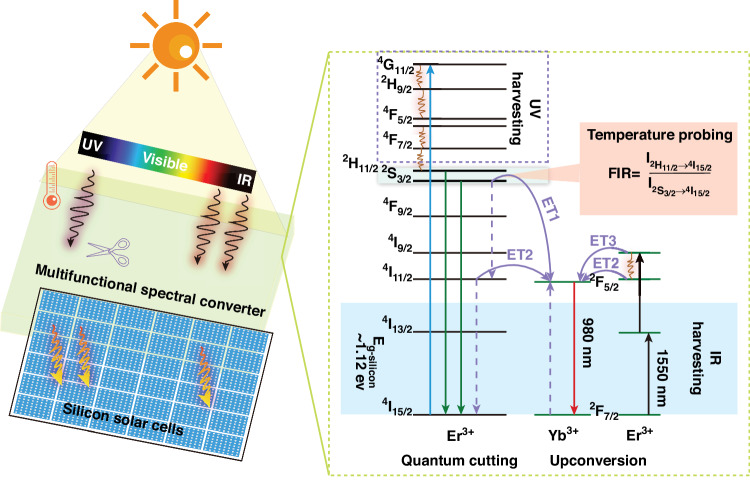
A schematic illustration of multifunctional NaY(WO_4_)2: Er^3+^/Yb^3+^ phosphors mounted on the front of silicon solar cells to enhance their performance. The zoom-in figure on the right provides a simplified representation of the energy levels of Er^3+^ and Yb^3+^ ions, along with the energy steps involved in the quantum cutting and upconversion processes. The ultraviolet (UV) harvesting for quantum cutting is highlighted in a dashed rectangular outline on the left part of the zoom-in figure, while the infrared harvesting within the silicon bandgap is emphasized with a light blue background on the left of the zoom-in figure. The reservoir ^2^H_11/2/_^4^S_3/2_ energy levels, crucial for accumulating photoexcited energy via multiphonon-assisted relaxations and providing fluorescence ratiometric ability for temperature probing, are highlighted in a cyan background. Solid straight lines in color (blue, green, and red) represent the radiative transitions, while solid curvy lines illustrate the involved energy transfer processes (ET1, ET2, and ET3). Dashed straight lines in purple and solid black lines represent the direction of energy transfer and the absorption of the excited photon, respectively. Dashed zig-zag arrows (in brown) illustrate the nonradiative multiphonon relaxation process

It has been established that, under 1550 nm excitation, singly Er^3+^-doped materials exhibit upconversion luminescence at 1000 nm, originating from the ^4^I_11/2_ → ^4^I_15/2_ transition. The ^4^I_11/2_ energy level is populated through a two-photon upconversion process, wherein a ground state absorption of the excitation photon populates the ^4^I_13/2_ energy level. Subsequently, energy transfer from another Er^3+^ ion at the ^4^I_13/2_ energy level can further elevate the Er^3+^ ion from the ^4^I_13/2_ energy level to the ^4^I_9/2_ energy level. This is followed by multiphonon-assisted relaxation, ultimately leading to the population of the ^4^I_11/2_ energy level. The efficient ET2 process found in quantum cutting process results in the intense emission of Yb^3+^ at 1000 nm through the ^2^F_5/2_ → ^2^F_7/2_ radiative relaxation. This is substantiated by the observation that the presence of Yb^3+^ ions results in 20-fold increase at 1000 nm, in comparison to the singly Er^3+^-doped NaY(WO_4_)_2_ phoshpors. Note that while the ET3 process is not explicitly mentioned, it cannot be ruled out as a contributor to the population of Yb^3+^ ions at the ^2^F_5/2_ energy level.

The intensity ratio of luminescence at 530/552 nm for the ^2^H_11/2_/^4^S_3/2_ reservoir energy levels has been well established to be very sensitive to local temperature change. The fluorescence intensity ratio (FIR) of ${I}_{{2\atop}H_{11/2}\to {4\atop}{I}_{15/2}}/{I}_{{4\atop}S_{3/2}\to {4\atop}{I}_{15/2}}$ can be expressed as^14^:

1 $${FIR}=\frac{{I}_{{2\atop }H_{11/2}\to {4\atop }{I}_{15/2}}}{{I}_{{4\atop }S_{3/2}\to {4\atop }{I}_{15/2}}}=N\exp \left(\frac{-\Delta E}{{kT}}\right)$$

where ${I}_{2{\rm{H}}11/2\to 4{\rm{I}}15/2}$ and ${I}_{4{\rm{S}}3/2\to 4{\rm{I}}15/2}$ are the integrated intensities of emission bands centered at 530 and 552 nm, respectively; *N* is a constant, *ΔE* is the energy gap between the thermally coupled energy levels ^2^H_11/2_ and ^4^S_3/2_, *k* is Boltzmann’s constant, and *T* is the absolute temperature. Precise temperature measurements were conducted within the range of 300–720 K, revealing a maximum absolute temperature sensitivity of ~0.01 K^−1^ at 443 K and maximum relative temperature sensitivity of 0.01 K^−1^ at 300 K. Given that solar cells typically operate at temperatures lower than 573 K (300 °C) to prevent damage, the developed NaY(WO_4_)_2_:Er^3+^/Yb^3+^ runs within this temperature range (300–573 K), exhibiting absolute and relative sensitivities approximating their maximum values. This observation aligns with the limited thermal quenching of the overall intensity of the ^2^H_11/2_/^4^S_3/2_, decreasing by only 25.8% when elevating temperature from 300 K to 720 K.

While various upconverting, quantum-cutting materials, and temperature sensors have been individually explored to enhance the performance of SSCs^15–19^, this study marks the first introduction of a multifunctional photon conversion material, NaY(WO_4_)_2_:Er^3+^/Yb^3+^, combining all three modalities. This innovative approach holds the potential for synergistic effects to maximize the efficiency of industrially produced SSC modules without altering their existing structures. The exceptionally high quantum cutting efficiency (173%) of ultraviolet light, impressive upconversion of sub-bandgap photons, and high-temperature sensitivity are attributed to the appropriate lattice phonon energy of the host material (900 cm^−1^). This phonon energy is sufficiently high to facilitate effective accumulation of harvested ultraviolet energy to the ^2^H_11/2/_^4^S_3/2_ reservoir energy level for quantum cutting and temperature sensing through multiphonon-assisted relaxations. Simultaneously, the lattice phonon energy is small enough (compared to the large energy gap of 3091 cm^−1^) between the ^2^H_11/2/_^4^S_3/2_ energy levels and the ^4^F_9/2_ energy level to prevent further nonradiative relaxations at the reservoir energy levels. Importantly, the relatively large lattice phonon energy, compared to hexagonal sodium yttrium fluoride (350 cm^−1^), plays a pivotal role in bridging an energy mismatch of ~2200 cm^−1^ in the first step ET 1 process to entail an efficient quantum cutting process.

Despite the significant progress made in this work, certain challenges still require further exploration. Firstly, there is a need for experimental confirmation or measurement of efficiencies for quantum cutting and upconversion processes, but quantifying the absorbance of lanthanide phosphors presents a challenge. Experimental determination of luminescence quantum efficiency will provide a solid basis for comparison among different luminescent materials and evaluation for practical applications. Secondly, lanthanides have narrow and limited absorption properties in both the infrared and ultraviolet ranges, ~10-fold narrower and 10,000 times lower than those of organic dye molecules^20^. This limitation poses a significant challenge for the practical application of lanthanide-based luminescent materials as spectral converters to enhance SSCs performance. However, coupling these phosphors with both infrared dyes to harvest light above 1100 nm and ultraviolet dyes to harvest light below 530 nm for sensitizing the upconversion and quantum-cutting processes holds great promise. This approach could potentially make these multifunctional phosphors more practical for bolstering the power conversion efficiency of SSCs beyond the Shockley-Queisser limit.
